# Nanofiller-Enhanced Soft Non-Gelatin Alginate Capsules for Modified Drug Delivery

**DOI:** 10.3390/ph14040355

**Published:** 2021-04-13

**Authors:** Sameer Joshi, Rajnish Sahu, Vida A. Dennis, Shree R. Singh

**Affiliations:** Center for NanoBiotechnology Research, Alabama State University, Montgomery, AL 36101, USA; sjoshi@alasu.edu (S.J.); rsahu@alasu.edu (R.S.); vdennis@alasu.edu (V.A.D.)

**Keywords:** capsules, non-gelatin, alginate, montmorillonite, drug delivery

## Abstract

Capsules are one of the major solid dosage forms available in a variety of compositions and shapes. Developments in this dosage form are not new, but the production of non-gelatin capsules is a recent trend. In pharmaceutical as well as other biomedical research, alginate has great versatility. On the other hand, the use of inorganic material to enhance material strength is a common research topic in tissue engineering. The research presented here is a combination of qualities of alginate and montmorillonite (MMT). These two materials were used in this research to produce a soft non-gelatin modified-release capsule. Moreover, the research describes a facile benchtop production of these capsules. The produced capsules were critically analyzed for their appearance confirming resemblance with marketed capsules, functionality in terms of drug encapsulation, as well as release and durability.

## 1. Introduction

The solid dosage form has retained its importance in the pharmaceutical industry due to easy production as well as handling and because it is the safest way of delivering the drug. Capsules are one of the solid dosage forms that are available in the market as soft and hard capsules. Soft capsules are the dosage form that is prepared as a single unit, and the hard capsules are usually a double unit dosage form. Depending on the preparation material, the soft capsules are classified as soft gelatin capsules and soft non-gelatin capsules. The soft gelatin capsules’ composition is gelatin, plasticizers, water, preservatives, coloring agents, opacifying agents, flavoring agents, and sweeteners [1].

Moreover, if the soft gelatin capsule is intended for intrinsic release, then the intrinsic coating could be an extra compositor of the capsule. The disintegration time for these capsules is faster compared to the disintegration time of non-gelatin capsules [2]. Although the soft gelatin capsule covers most of the soft capsule market, the soft non-gelatin capsules are also gaining consumer interest. This is due to a variety of reasons, such as consumer choice [3], the reaction of unmodified gelatin with aldehydes [4,5], problems in intrinsic release, and temperature sensitivity [5].

Alginate, available as alginic acid, is the product obtained from brown algae. The use of alginate in research in recent years has dramatically increased due to its biocompatibility [6] and easy availability. Alginate has been reported as a major component in studies such as dosage form preparation [7] as well as tissue engineering [6]. The mechanical and chemical stability of the capsules is essential in delivering drugs [8]. Depending on the type of material used in the capsule preparation, as well as the location of the delivery and the on-site pH, the release of the drug from the capsule may vary. On the other hand, nanofiller-enhanced gels are one of the novel types and are mechanically stronger than conventional gels [9]. Depending on the type of application, different nanofillers, such as montmorillonite (MMT) [10], bentonite [11], laponite [12], etc., are being used in research. In recent years, researchers have mentioned that clays can be used as catalysts [13], adsorbents [11,14], metal chelating agents [15], as well as polymer nanocomposites [9,13]. The MMT is a product of volcanic ash [16] and is usually described as a layered structure made of aluminosilicate layers, which are silica or aluminum stacks with Na^+^ counterions [17]. The structure of clay is a sandwich of the octahedral sheet between two tetrahedron sheets, and the layer distance between two aluminosilicate layers is around 10Å or 1 nm. Homogenous dispersion of MMT into the gel provides excellent strength to the gel. 

The quality of the dosage form is determined by its safety, stability, efficacy, and patient compliance [8]. The research in this study describes the alginate capsules with or without MMT that were prepared and tested for their appearance, size uniformity, shape uniformity, content uniformity, mass variation, stability, disintegration, the pattern of capsule disintegration, or the in vitro release of the active ingredient. 

## 2. Results 

### 2.1. Optimization of Material Concentration

**Figure 1:**
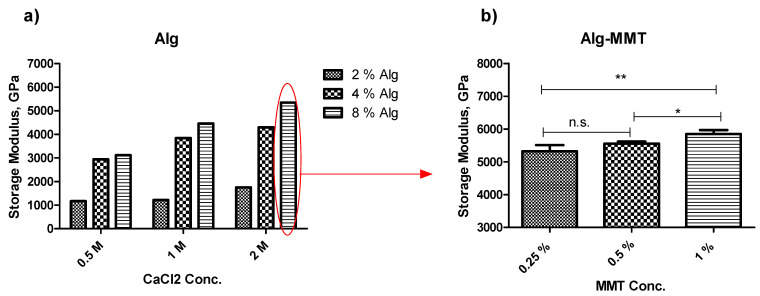
Optimization of component concentration. (**a**) Optimization of concentration of alginate and the cross-linker CaCl_2_ (*n* = 1). (**b**) Optimization of MMT concentration (*w/v*) in the capsule with optimized alginate and CaCl_2_ concentration (red circled in Figure 2a) (*n* = 3, ± SD). (Signs * and ** represents a significant difference < 0.05 and 0.01, respectively, and n.s. represents “not significant”.)

Optimization of material concentration was performed to determine the best working concentration of alginate as well as CaCl_2_. When three different concentrations of the alginate were tested against three concentrations of CaCl_2_, better storage modulus was achieved at higher concentrations of alginate as well as CaCl_2_ (Figure 1a). Moreover, optimization of the MMT concentration was performed using the optimized concentrations of alginate and CaCl_2_ (Figure 1b). However, there was no significant difference observed within the capsules with varying concentrations of MMT. 

### 2.2. Precision Production and Effect of MMT Addition

Soft capsules are available in a range of sizes. However, the standard industrial ones are manufactured in a range from 000 to 5 [3,18]. In this study, the capsules prepared were of “00” size (Figure S1, Supplementary Materials). On the other hand, the shape of the soft capsule can be an oval, spherical, ovoid, tube, pear, etc. The soft capsules of alginate in this study appeared to be oval. In addition to the oval shape, a ring formation was observed at the junction after the cross-linking. The nanofiller-enhanced capsules appear to be intact and uniform compared to the alginate alone. 

**Figure 2:**
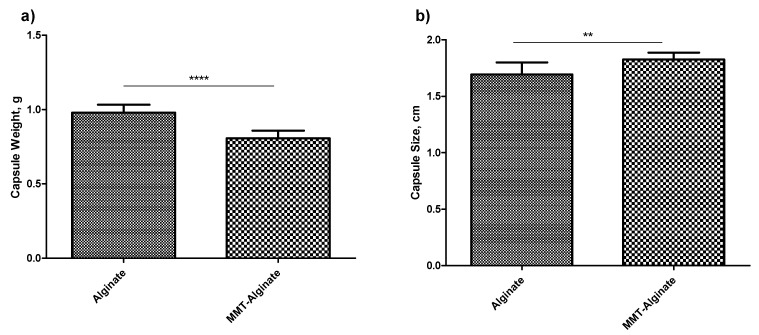
Effect of addition of MMT. (**a**) Comparison of weight variation between alginate capsule and MMT-enhanced alginate capsules (*n* = 12, ±SD). (**b**) Comparison of size variation between the alginate capsules and MMT-enhanced alginate capsules (*n* = 12, ±SD). Signs **** and ** represents a significant difference.

To confirm the robustness of the method of preparation as well as to know the effect of the addition of MMT to alginate, a mass and size variation analysis was performed. For this purpose, twelve alginate capsules with or without MMT were accurately weighed for this analysis. In both cases, the standard deviation was less than 0.5. However, the addition of MMT significantly reduced the weight of the capsules (*p* < 0.05, *t*-test) (Figure 2a). However, with regards to the size, the alginate capsules produced were significantly smaller (*p* < 0.05, *t*-test), on average, than the MMT-enhanced alginate capsules (Figure 2b). Moreover, the standard deviation of the size of 12 alginate capsules was more than the MMT-enhanced alginate capsules. 

### 2.3. Stability Study (90 Days)

**Figure 3:**
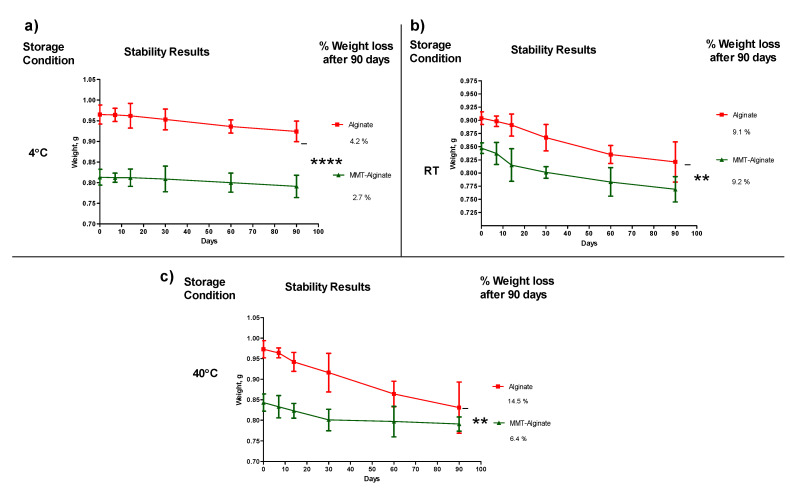
Three months of stability study of the alginate and MMT-enhanced alginate capsules. The study was performed in three different temperatures: (**a**) 4 °C, (**b**) room temperature (RT), and (**c**) elevated temperature 40 °C. The stability/significant difference was judged based on the capsule weight at 90 days (*n* = 4, ± D). Signs **** and ** represents a significant difference.

The stability of the capsules was measured based on weight loss upon storage. Each capsule was accurately weighed at a given interval, and at the end of 90 days, the % weight loss was calculated. The weight loss was less than 10% in all storage conditions except for the alginate capsule without MMT stored at 40 °C. The storage temperature of 4 °C has shown the least weight loss among all the temperatures (Figure 3a). In the case of capsules enhanced with MMT, at all temperature conditions, the amount of weight loss after 90 days was significantly lower (*p* < 0.05, *t*-test) compared to the capsule without MMT (Figure 3a–c).

### 2.4. Percent Drug Encapsulation and Content Uniformity

**Figure 4:**
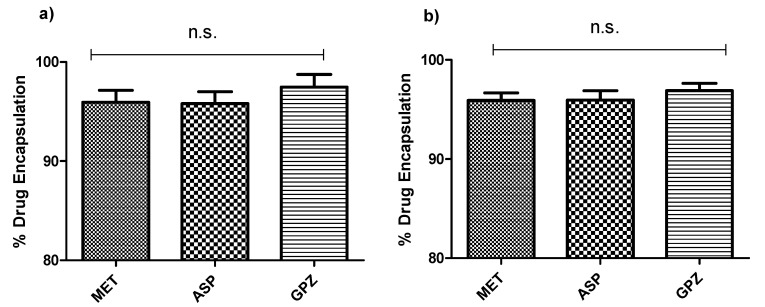
Three model drugs—metformin (MET), Aspirin (ASP), and Glipizide (GPZ)—and their percent drug encapsulation determined spectrophotometrically (*n* = 4, ±SD). (n.s. = not significant). (**a**) Alginate capsules without MMT and (**b**) alginate capsules enhanced with MMT. The SD ± % represents the content uniformity of the encapsulated drug in the capsules.

The drug encapsulation was determined spectrophotometrically by determining the concentration of the tablet dissolved. For both types of capsules (alginate and alginate-MMT), it was observed that % encapsulation was over 90% for all the three model drugs: MET, ASP, and GPZ. Moreover, the % encapsulation slightly varied with all the three drugs, but there was no significant difference observed for the percent encapsulation of three drugs (One-way ANOVA, Tukey post-test) (Figure 4).

### 2.5. Behavior of Capsule in Disintegration Media with Different pH 

**Figure 5:**
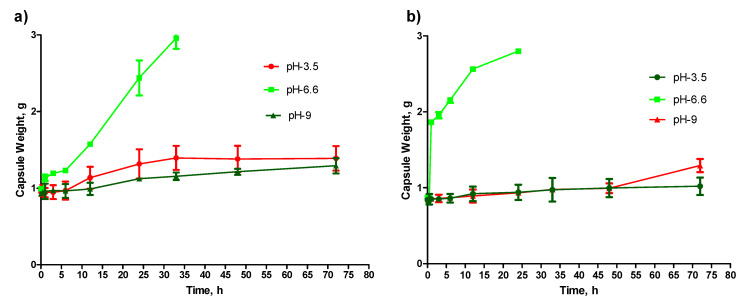
Analysis of capsules’ behavior in different media (pH 3.5, 6.6, and 9). (**a**) Weight of alginate capsule recorded at a different interval. (**b**) Weight of MMT enhanced alginate capsule recorded at a different interval (*n* = 3, ±SD).

The purpose of this study was to know the effective media and the pH to disintegrate the capsule. Therefore, the effect of pH on the structure and mass of the capsule was observed in this section of the study. Capsules were found unaffected at strong acidic as well as strong basic pH. However, at a slightly acid pH, both capsules started disintegrating. Moreover, it was also observed that capsules enhanced with MMT tend to disintegrate significantly faster (*p* < 0.05) than capsules without MMT (Figure 5a,b). 

### 2.6. Real-Time Imaging of Capsule Disintegration and Drug Release

**Figure 6:**
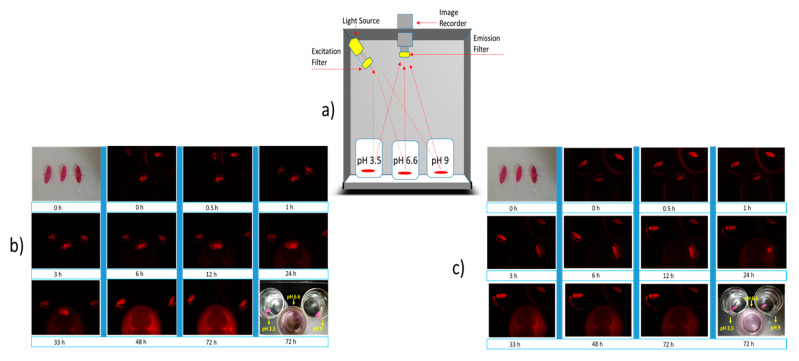
Real-time imaging of tablet disintegration in media with different pH. DilC-18 stain (red) was encapsulated into the capsule to study the pattern of drug release. (**a**) Imaging of alginate capsule at different time intervals. (**b**,**c**) Imaging of alginate and MMT-enhanced alginate capsule, respectively, at different time intervals.

The pH of the human digestive system varies throughout the system. In the stomach, the pH is usually ~3.5, but starting from the proximal small intestine to the descending colon, the pH ranges between 6.5 and 7.5 [19]. Therefore, capsules were tested in physiological pH media as well as in elevated pH media to study the effect of different pH media on the capsules. The purpose of using the fluorescent dye Dil-C-18 for this experiment was to understand the possible pattern of capsule disintegration and drug release at different pH. The result of this study matches the findings in Section 4.5. There was no effect on capsule appearance observed at pH 3.5 and 9. However, the capsule gradually becomes swollen, absorbing the aqueous media at pH 6.6 (Figure 6a,b). Complete dissolution was observed for a capsule with MMT and without MMT after 24 hrs and 33 hrs, respectively. Again, similar to the findings ni Section 4.5, the capsule enhanced with MMT tends to disintegrate faster than the capsule without MMT. 

### 2.7. In Vitro Drug Release 

**Figure 7:**
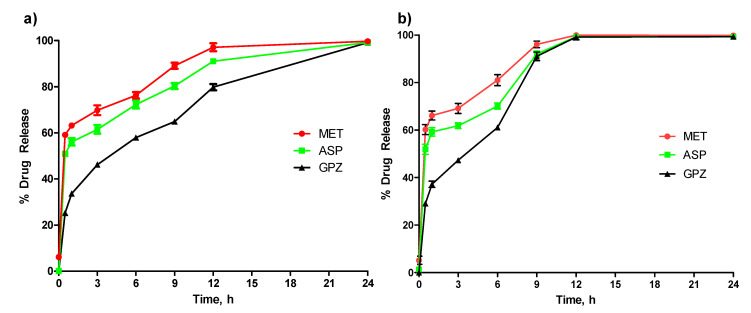
Analysis of in vitro drug release in the media resembling intrinsic pH 6.6. Three model drugs—MET, ASP, and GPZ—encapsulated in (**a**) alginate capsule and (**b**) MMT-enhanced alginate capsule (*n* = 3, ±SD).

After optimizing the component concentration and drug release media, an in vitro drug release study was performed at pH 6.6. In this study, three drugs of different solubility were tested, with MET as highly soluble and GPZ as least soluble. For both the capsules, the order of drug release was MET > ASP > GPZ (Figure 7a,b). However, although the order of drug release was similar in both the capsules, the capsule with MMT released the drug faster than without MMT (Figure 7b).

### 2.8. Microscopic Morphology

**Figure 8:**
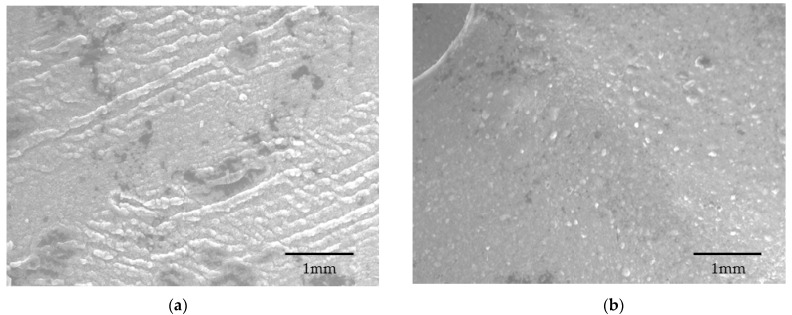
Microscopic evaluation of capsule surfaces. (**a**) Alginate capsule without MMT and (**b**) alginate capsule with MMT. The imaging was performed at ×500 magnification.

Microscopic elucidation clearly shows that both the capsules have a porous surface (Figure 8a,b). However, the MMT-enhanced capsules (Figure 8b) appear ordered/uniform compared to the capsule without MMT (Figure 8a). 

## 3. Discussion

This manuscript reports a facile method for the production of alginate capsules (Figure 1). In general, the development of the product starts with the optimization of the component concentration; therefore, this research begins with the optimization of the concentration of alginate as well as the cross-linker CaCl_2_. Note that as the concentration of alginate and CaCl_2_ increases, the storage modulus increases too (Figure 1). However, it was reported that MMT at concentrations of more than 1% *w/v* gives bone-like rigidity to the structure as well as increases the pore size [20]. Therefore, three different concentrations of MMT were tested, and the 1% *w/v* concentration of MMT with the best G’ was chosen to achieve maximum possible rigidity along with the pore size suitable to avoid delayed release of the active ingredient. 

To determine the reproducibility of the method of capsule production, the uniformity in mass as well as the size and shape of the soft capsule can be recorded. As per the recommendations of the United States Pharmacopeia (USP), twelve capsules were accurately weighed individually and tested for uniformity in the mass, and there was no significant difference observed in the masses of individual capsules (SD < 0.5%). However, there was a significant weight difference (*p* < 0.05) observed between the alginate capsules and the nanofiller-enhanced alginate capsules (Figure 2). The lower weight of the nanofiller-enhanced capsule shows that the alginate was replaced by the nanofiller, giving a possible indication of an increased number of pores as well as pore size compared to the alginate capsules without nanofillers. Analyzing the size and shape is another aspect of judging the method’s robustness. In both cases, the size of the capsules was found resembling the size “00”. However, the capsules with MMT are not only distinct in color, but also in overall appearance (Figures S1 and S3).

The vital characteristic of any soft-gel capsule is its capacity to stay hydrated at any given temperature. Therefore, the temperature-dependent weight loss was studied during the stability testing. The major findings of this study are less than 10% weight loss (with respect to the initial weight) and significant difference in weight loss of alginate capsules compared to the alginate–MMT capsules. This confirms that the alginate itself has the capacity to stay hydrated at any given temperature and addition of MMT to this material reduces the dehydration of the capsules, hence less weight loss.

Both the capsules are shown to have resistance to high acid and basic environments (Figure 6a,b), and this finding is particularly significant when designing the dosage form for enteric drug release. As mentioned earlier in this report, the region’s pH from the proximal small intestinal to the large intestine is between 6.5 and 7.5. Both the capsules were found disintegrating at pH 6.6 (Figure 6a,b). Therefore, the release of the drug in the intestinal region can be achieved. This is particularly very important for drugs like MET and GPZ that have good absorbance in the intestinal region [21,22]. During this study, it was observed that the nanofiller-enhanced capsules disintegrate faster than the alginate capsule without nanofiller (Figure 6a,b). The possible logic behind this is the porous structure of the MMT-enhanced capsules that makes the media diffuse into the capsule faster. This faster diffusion of the media causes quicker pH-dependent oxidation of alginate chains, where the degree of oxidation is higher at pH 6.6 [23]. Therefore, the MMT enhanced capsule disintegrates faster. 

**Figure 9:**
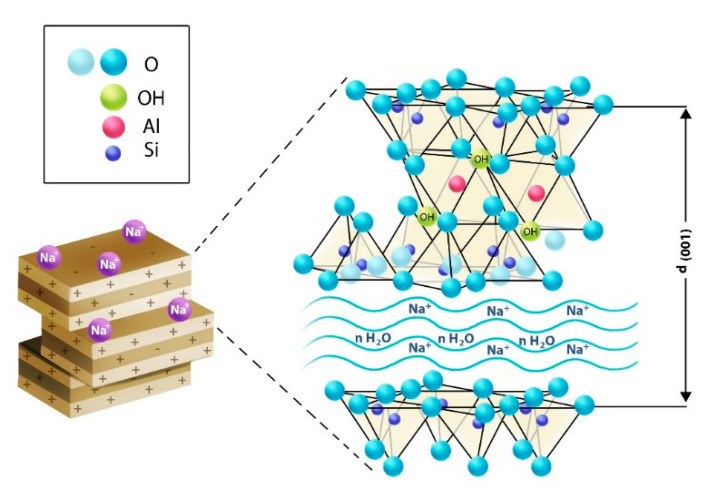
Schematic representing the structure of MMT, including functional groups and possible intercalation between the plates.

The SEM analysis of both the capsules confirms this statement. The MMT-enhanced capsule was found porous under SEM observation [24] (Figure 8a,b), and the reason for this porosity is the presence of MMT, which develops a porous structure through intercalation and exfoliation (Figure 9) of the MMT plates [24]. 

**Figure 10:**
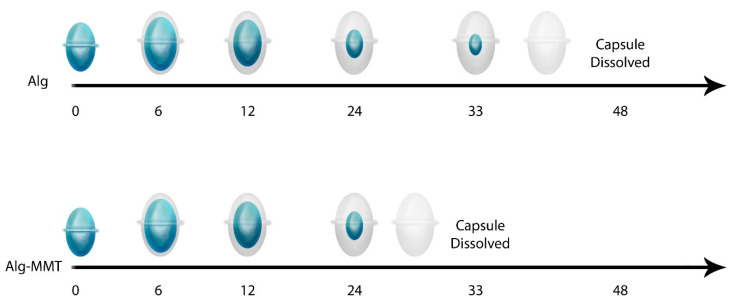
Based on the real-time imaging (Figure 7), capsule disintegration pattern at specific time-points.

With the aim of elucidating the possible pattern of drug release, capsules were loaded with a fluorescent dye, and the real-time capsule disintegration was observed under the fluorescence microscope. Both the capsules have a similar way of disintegration, that is, the absorption of water, with swelling leading to disintegration (Figure 10). 

Similar to the findings in Section 4.5, the nanofiller-enhanced capsules encapsulated with fluorescent dye disintegrated faster than the capsule without nanofillers. This study also confirmed the suitability of these capsules for intrinsic drug release, as there was no drug release observed into the strong acid or basic media (Figure 6a,b). Not only with the Dil-C dye, but also with another drug, the pattern of drug release is that the MMT-enhanced capsule released drug faster than the capsule without MMT (Figure 7a,b). The in vitro study also confirmed that drug release is not only affected by the inclusion of MMT, but also by the solubility of the drug. Among the three drugs tested, MET was the most soluble and was released faster than GPZ, the least soluble drug. This suggests that depending on the required dosage of the drug as well as the time to deliver the drug, the capsule can be designed with or without MMT. 

One of the most critical aspects of the dosage form is its stability. The stability of the capsules with MMT was significantly better (*p* < 0.05) than the capsules without MMT. The possible reason behind this is that MMT holds water through the intercalation of water between MMT plates. Moreover, as mentioned earlier, the MMT must have replaced the volume of alginate in the capsule, and thus not only was the weight of the capsule low, but there was also less alginate present in the capsules enhanced with the MMT. Therefore, MMT-enhanced capsules are shown to have less weight loss during storage at any temperature.

## 4. Materials and Methods

### 4.1. Materials 

Alginic acid and calcium chloride were of Acros Organics, purchased from Fisher Scientific, Fair Lawn, NJ, USA. MMT was of Alfa Aesar, purchased from Fisher Scientific, Fair Lawn, NJ, USA. For the preparation of the buffer that was needed for the capsule disintegration and drug release, sodium citrate, citric acid, sodium carbonate, and sodium bicarbonate were purchased from Fisher Scientific, Fair Lawn, NJ, USA. The model drugs metformin (MET), acetyl salicylic acid also known as aspirin (ASP), and glipizide (GPZ) were purchased from Sigma-Aldrich, USA. Hydrochloric acid used to adjust the buffer pH was purchased from Sigma-Aldrich, USA. The water used in each experiment was of Milli-Q grade, and all the chemicals were of analytical grade.

### 4.2. Preparation of Capsule

**Figure 11:**
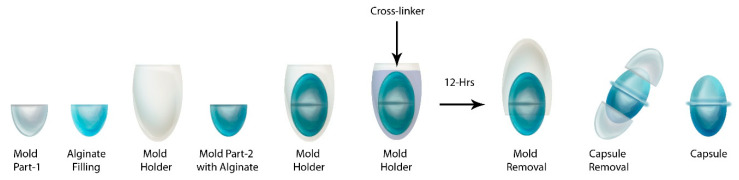
Schematic representing the process of alginate capsule production. The process included filling the mold with alginate with or without MMT, placing the 2-parts mold into the mold holder, and addition of cross-linker followed by removal of mold after 12 h.

The capsules were prepared using the molds of capsule size “00”. Briefly, alginate with or without MMT was injected into the two parts of the capsule mold. Then, these two parts were joined into a mold holder. The mold holder was approximately 1 mm wider than the mold. This 1 mm gap was used to expose the alginate to the CaCl_2_ that was added covering the mold. This whole assembly was placed on a 3-D rotator for 12 h, followed by removal of capsules from the mold (Figure 11). The respective model drug was mixed with the alginate prior to mold filling. The procedure was performed at room temperature and

### 4.3. Optimization of Component Concentration

Capsules were tested for their appearance (size and shape) and storage modulus (G’) to optimize their major component concentration. To optimize the concentrations of alginate and the cross-linker, four different concentrations of alginate were tested against four different concentrations of CaCl_2_. The storage modulus was determined using the rheometer (Discovery HR-1, TA-instruments, USA). Increasing concentration of MMT increases pore size, and 2% *w/v* MMT concentration can give strength equivalent to the bone [20]; considering this, concentrations up to 1% *w/v* MMT were chosen to optimize the final concentration. During this part of the study, individual capsules with optimized alginate and CaCl_2_ concentration were prepared; these individual capsules were enhanced with 0.25, 0.5, or 1% *w/v* MMT; and the G’ was recorded to study the effect of inclusion on MMT on the capsule structure. 

### 4.4. Appearance and Mass Uniformity

After optimizing the component concentration, in this section of the study, the capsules were examined for the uniformity in the size, shape, and overall look of the capsule. To test the mass uniformity, twelve capsules of the 8% alginate crosslinked with 2M CaCl_2_ and with or without 1% *w/v* MMT were prepared. Each demolded capsule was accurately weighed and compared for the mass variation and size variation. 

### 4.5. Stability of the Capsules

Upon storage, no change in the soft capsule weight is a sign of its stability. An increase in the weight can be from moisture absorption, and a decrease in the weight indicates dehydration. Stability is achieved if the material varies with storage temperature as well as packaging. In this study, the stability of both alginate and nanofiller-enhanced alginate capsules was tested over a period of 90 days. Three different temperatures (40 °C, 4 °C, and room temperature) inside the airtight glass vial were considered for storage during this study. Capsules were weighed accurately on 0, 1, 7, 15, 30, 60, and 90 days.

### 4.6. Scanning Electron Microscopy

The cross section morphology of the capsules was examined by a scanning electron microscope (SEM) (JEOL technology, Peabody, MA, USA). The imaging was performed at a voltage of 10 kV with a current of 5 mA. Before the analysis, the samples were sputter-coated with gold to a thickness of 5 nm. 

### 4.7. Drug Encapsulation

Three drugs—MET, ASP, and GPZ—were chosen as a model drug. Ten milligrams of each model drug was used to be encapsulated into the capsule. The capsule encapsulating the model drug was disintegrated and dissolved into the appropriate media to ensure the total drug release was achieved. The concentration of the drug was determined using UV/Visible spectrophotometry assay, and the quantification was done at 232 nm, 2270 nm, and 2232 nm, respectively, for MET, ASP, and GPZ. Further, the percent encapsulation was determined using Equation (1), where *ai* is the initial amount and *af* is the amount determined after dissolving the capsule.

(1) $$\%\;\mathrm{Encapsulation}=\frac{af}{ai}\times 100$$

### 4.8. Selection of Capsule Disintegration Medium 

Capsules with Dil-C dye (Figure S2, Supplementary Materials) were tested for disintegration prior to drug encapsulation. For this purpose, buffers of 3 different media were chosen: citrate buffer (pH 3.0), citrate buffer (pH 6.6), and bicarbonate buffer (pH 9). Briefly, into a 200 mL beaker having a buffer, a capsule was added and imaged (Section 4.6) at intervals of 0, 0.5, 1, 3, 6, 12, 24, 33, 48, and 72 h. The experiment was performed three times to confirm the finding. 

### 4.9. Fluorescence Imaging 

A fluorescent dye (Dil-C_18_) was considered as a drug and encapsulated into the capsules. The dye was mixed with alginate prior to its injection into the mold. To confirm the results from Section 2.8 and to know the capsule disintegration pattern, a capsule was added into a beaker having the release media of different pH in every beaker, as mentioned in Section 2.8. The beakers were kept at physiological temperature in a dark chamber equipped with fluorescence excitation/emission filters and a charged-coupled device (CCD) camera attached on top to capture the image (iBox Scientia UVP imaging system, Analytic Gena, Upland, CA, USA) as shown in Figure 8. Capsules with encapsulated fluorescence dye were then imaged at time intervals of 0, 0.5, 1, 3, 6, 12, 24, 33, 48, and 72 h.

### 4.10. In Vitro Drug Release 

Capsules with a different drug of different solubility and with or without MMT were prepared for this study. The drug was mixed with alginate prior to its injection into the mold. A capsule was placed into a beaker having the 1.0 L release media in every beaker and shaken at a speed of 150 rpm. The beakers were kept at physiological temperature, and samples were withdrawn at the intervals of 0, 0.5, 1, 3, 6, 12, 24, 33, 48, and 72 h. The amount of drug released was determined using UV–Visible spectrophotometry (Nanodrop, Thermo Scientific, Waltham, MA, USA). Sample absorbance was recorded at a predetermined wavelength of 233 nm, 265 nm, and 233 nm for MET, ASP, and GPZ, respectively [25,26]. 

### 4.11. Statistical Tool 

Unless stated otherwise, the results were calculated as mean ± standard deviation (SD). A T-test and ANOVA followed by Tukey post hoc analysis was performed for comparison, and significance was acknowledged for *p* values less than 0.05. All the calculations were made using Prism 6 (GraphPad Inc., San Diego, CA, USA).

## 5. Conclusions

This research reports a facile method of production of alginate capsules. The capsules molded in this research were oval, but the material can be molded in any shape as per the requirement. Inclusion of MMT has advantages such as intact shape, faster release, and better stability. However, if delayed drug release is required, then MMT can be excluded. This research also reports the pattern of drug release from the capsules, which may help in the further development of the formulation. The drug’s solubility is a very important aspect of drug delivery, and the capsule can be used to deliver low solubility drugs such as GPZ. The most important feature of these alginate capsules is that they are resistant to a hostile gastric environment. Therefore, the capsules are the best fit to carry drugs that are prone to degradation in this gastric environment and have better absorbance in the intestinal region. However, further developments in the formulation, such as the different shapes of the capsule and different concentrations, as well as the type of nanofillers, could make the product marketable. 

## Data Availability

The data reported in this study are available in this manuscript, supplementary file, or from the corresponding author upon requests.

## Supplementary Materials

The following are available online at https://www.mdpi.com/article/10.3390/ph14040355/s1, Figure S1: Appearance of the alginate capsule. A red window indicates the alginate capsule is size ’00’ and a ring formation observed at the middle of the oval capsule, Figure S2: Dil-C (Fluorescent dye encapsulated capsules.
