# Supplementary material for: Nanofiller-Enhanced Soft Non-Gelatin Alginate Capsules for Modified Drug Delivery
Source: Pharmaceuticals (Basel). 2021 Apr 13;14(4):355. doi: 10.3390/ph14040355 (PMC8069733; doi:10.3390/ph14040355)
Supplement: Supplementary file 1 [file pharmaceuticals-14-00355-s001.pdf]

<u>Supplementary Information</u>

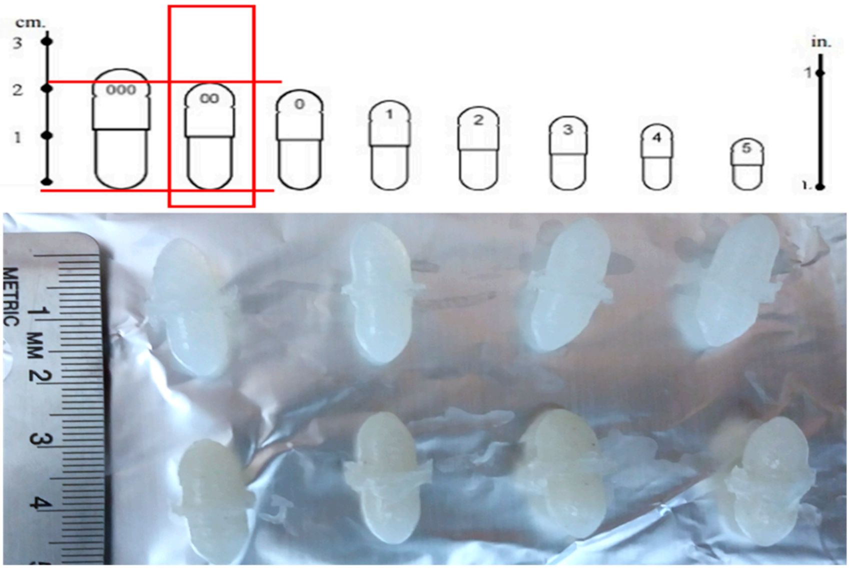

Figure S1 Appearance of the alginate capsule. A red window indicates the alginate capsule is size '00,' and a ring formation observed at the middle of the oval capsule.

a) **b)**

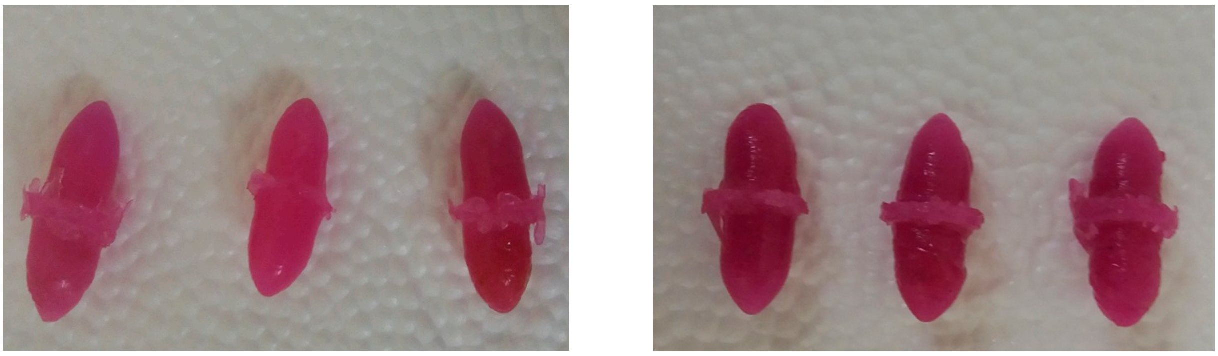

Figure S2 Dil-C (Fluorescent dye encapsulated capsules). a) Alginate capsules with Dil-C dye. b) Alginate-MMT capsules with Dil-C dye. The Capsules with MMT looks intact compared to one without MMT.
